# Artificial intelligence driven Mid-IR photoimaging device based on van der Waals heterojunctions of black phosphorus

**DOI:** 10.1515/nanoph-2024-0613

**Published:** 2025-02-13

**Authors:** Ziqian Wang, Huide Wang, Chen Wang, Yushuo Bao, Weiying Zheng, Xiaoliang Weng, Yihan Zhu, Yi Liu, Yule Zhang, Xilin Tian, Shuo Sun, Rui Cao, Zhe Shi, Xing Chen, Meng Qiu, Hao Wang, Jun Liu, Shuqing Chen, Yu-Jia Zeng, Wugang Liao, Zhangcheng Huang, Haiou Li, Lingfeng Gao, Jianqing Li, Dianyuan Fan, Han Zhang

**Affiliations:** State Key Laboratory of Radio Frequency Heterogeneous Integration, International Collaborative Laboratory of 2D Materials for Optoelectronics Science and Technology, Institute for Advanced Study in Nuclear Energy & Safety, Interdisciplinary Center of High Magnetic Field Physics of Shenzhen University, College of Physics and Optoelectronic Engineering, Shenzhen University, Shenzhen 518060, China; College of Chemistry and Chemical Engineering, Ocean University of China, Qingdao 266100, China; International Collaborative Laboratory of 2D Materials for Science and Technology of Ministry of Education, Institute of Microscale Optoelectronics, 47890 Shenzhen University, Shenzhen 518060, Guangdong, China; College of Electronic and Information Engineering, 47890 Shenzhen University, Shenzhen 518060, China; Key Laboratory of Optoelectronic Devices and Systems of Ministry of Education and Guangdong Province, College of Physics and Optoelectronic Engineering, 47890 Shenzhen University, Shenzhen 518060, China; School of Computer Science and Engineering, Macau University of Science and Technology, Cotai, Macau; School of Physics & New Energy, Xuzhou University of Technology, Xuzhou 221018, China; School of Electronic Engineering, Chengdu Technological University, Chendu 611730, China; State Key Laboratory of Radio Frequency Heterogeneous Integration, College of Mechatronics and Control Engineering, 47890 Shenzhen University, Shenzhen 518060, P.R. China; State Key Lab of Integrated Chips and Systems, Frontier Institute of Chip and System, Fudan University, Shanghai 200433, China; Guangxi Key Laboratory of Precision Navigation Technology and Application, Guilin University of Electronic Technology, Guilin 541004, China; College of Material Chemistry and Chemical Engineering, Key Laboratory of Organosilicon Chemistry and Material Technology, Ministry of Education, Key Laboratory of Organosilicon Material Technology, Hangzhou Normal University, Hangzhou 311121, Zhejiang, P.R. China

**Keywords:** black phosphorus, mid-infrared, photodetectors, single-pixel imaging, artificial intelligence

## Abstract

Mid-infrared (Mid-IR) photodetection and imaging are pivotal across diverse applications, including remote sensing, communication, and spectral analysis. Among these, single-pixel imaging technology is distinguished by its exceptional sensitivity, high resolution attainable through the sampling system, and economic efficiency. The quality of single-pixel imaging primarily depends on the performance of the photodetector and the sampling system. Photodetectors based on black phosphorus (BP) exhibit low dark current, high specific detectivity (*D* ^*^), and room-temperature operability. Artificial intelligence (AI)-assisted sampling systems feature efficient and intelligent data reconstruction capabilities. In this work, we demonstrate an AI-driven black phosphorus (BP)/molybdenum disulfide (MoS_2_)/hexagonal boron nitride (hBN) heterojunction for Mid-IR photodetection and imaging. By optimizing the thickness of the heterojunction, the quality of the interface, and the AI algorithm, we achieved high-performance Mid-IR photodetection and imaging. Specifically, the photodetector has a responsivity of 0.25 A/W at a wavelength of 3,390 nm, an extremely high *D* ^*^ of 3.7 × 10^9^ Jones, a response speed as low as 7 ms, and after AI optimization, the image contrast ratio has been improved from 0.227 to 0.890. At the same time, the sampling rate requirement can be reduced to 25 %. Our research indicates that the efficient combination of BP heterojunction photodetectors and AI technology is expected to accelerate the development of Mid-IR photodetectors and imaging systems.

## 1 Introduction

Mid-infrared (Mid-IR) photodetection refers to the technology of detecting and analyzing optical signals in the Mid-IR wavelength band, typically ranging from 3 to 5 μm [1], [2]. Mid-IR light has characteristics such as strong penetration and the ability to identify different molecular vibrations [3], making Mid-IR photodetectors widely applicable in various fields, including remote sensing monitoring, free-space optical communication, military reconnaissance, environmental monitoring, chemical analysis, and medical diagnostics [4]. Imaging systems based on Mid-IR photodetectors mainly consist of single-pixel imaging and focal plane array imaging [5], [6], [7], [8]. Among these, single-pixel imaging, due to the lack of device uniformity issues, features high sensitivity, low noise, large bandwidth, and low cost [9], [10]. A single-pixel imaging system generally comprises a spatial light modulator and a photodetector, which perceives the light intensity information of continuous spatial positions and displays the target image using the collected two-dimensional (2D) data. The image quality depends on the performance of the photodetector, modulator, and sampling system [5]. Currently, Mid-IR photodetectors are mainly manufactured using materials such as mercury–cadmium–telluride (MCT) [11], quantum wells [12], quantum dots [13], [14], type II superlattices [15], and silicon–germanium–tin [16]. The performance of these photodetectors is limited by complex manufacturing processes, low-temperature or ultra-low-temperature cooling requirements, and stability issues [17]. Therefore, the development of new types of Mid-IR photodetectors and sampling systems will promote the advancement of the field of Mid-IR single-pixel imaging.

On the one hand, 2D materials such as graphene [18], [19], BP [20], [21], As_*x*_P_1−*x*_ [22], [23], [24], [25], Te [26], [27], [28], PtSe_2_ [29], [30], [31], PdSe_2_ [32], [33], PtTe_2_ [34], and PdTe_2_ [35] have shown potential in Mid-IR photodetector applications because of their unique electrical and optical properties. The atomic thickness of 2D materials and the absence of surface dangling bonds endow them with advantages such as strong light–matter interaction, high internal quantum efficiency (IQE), low noise, and ease of heterostructure construction [36], [37], [38]. High-performance heterojunction Mid-IR photodetectors based on BP/MoS_2_ [39], [40], [41], graphene/BP [42], HgCdTe/BP [43], Te/graphene [44], and PtTe_2_/Si [45] have been reported. In addition, 2D materials also have characteristics such as high electron/hole mobility, in-plane anisotropy, and tunable bandgaps, making them promising for high-speed photodetection, polarization-sensitive photodetection, and other fields [17], [46]. On the other hand, in the sampling system of the single-pixel imaging system, due to the limitations of sampling speed, sampling accuracy, spatial information of the object, and image reconstruction, enhancing the performance of the sampling system helps to improve the final image quality [5]. To enhance the performance of the sampling system, optimizing the image reconstruction algorithm is a main research direction, such as using optimized reconstruction algorithms to improve image quality based on Hadamard [47], Fourier [48], and wavelet basis models [5]. Furthermore, compared with the above traditional algorithm models, AI mainly optimizes the image reconstruction process through deep learning models, which can more effectively improve image quality and efficiency [49]. Specifically, AI uses an “image-to-image” approach to denoise and enhance high-noise images, achieving a direct mapping from single-pixel measurements to high-quality images, thereby improving reconstruction efficiency and quality.

In this work, we selected the BP/MoS_2_/hBN heterojunction as a Mid-IR photodetector to achieve a wide spectral detection range. By controlling the thickness of the heterojunction and optimizing the interface quality, we obtained a Mid-IR photodetector that has low room-temperature noise and high sensitivity. At *V*_ds_ = −1.5 V, the room-temperature dark current is as low as 14.4 nA, the equivalent noise power (NEP) is as low as 10.57 pW/Hz^1/2^, the response band covers 405 nm–3,390 nm, the responsivity reaches 0.25 A/W at 3,390 nm, the specific detectivity (*D* ^*^) is as high as 3.7 × 10^9^ Jones at 3,390 nm, and the response speed is less than 7 ms at 3,390 nm. Concurrently, we constructed a Mid-IR single-pixel imaging system, achieved single-pixel imaging, and demonstrated the imaging effect. Through AI algorithm processing, we significantly enhanced the recognizability of the imaging, with the image contrast ratio improving from 0.227 to 0.890. Moreover, data with a low sampling rate density (as low as 25 %) can still achieve good imaging results, which significantly benefits the improvement of imaging speed and reduction of costs. Our research indicates that by combining advanced new photodetectors with AI technology, it is possible to develop Mid-IR imaging systems with superior performance and lower costs.

## 2 Results and discussion

**Figure 1:**
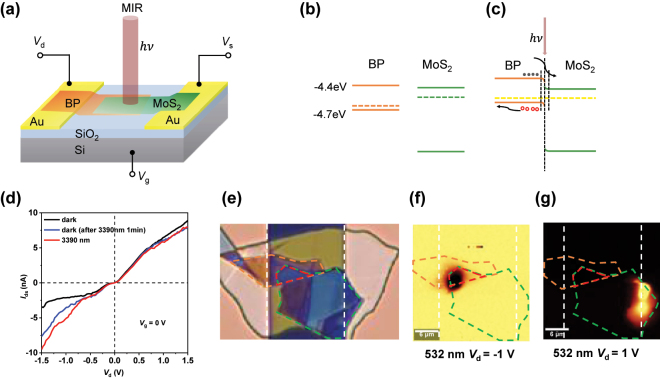
The structural schematic, band diagram, Mid-IR response curves, and photocurrent mapping images under different bias voltages of the BP/MoS_2_/hBN heterojunction device. (a) The structural schematic of the device, which from bottom to top consists of a silicon substrate, a silicon dioxide layer, BP, MoS_2_, and hBN. (b), (c) The band structure diagram of the device under Mid-IR illumination. (d) The IV_d_ curves of the device at 3,390 nm, depicting the dark current, the dark current measured immediately after 1 min of continuous illumination at 3,390 nm, and the photocurrent at 3,390 nm. (e) The optical micrograph of the device under an optical microscope, where the orange dashed line indicates BP, the green dashed line indicates MoS_2_, and the red dashed line indicates the heterojunction region. (f) The photocurrent mapping image of the device under a −1 V bias. (g) The photocurrent mapping image of the device under a +1 V bias.

Figure 1(a) illustrates the structural diagram of the BP-MoS_2_ heterojunction, where the laser is applied either directly or through an optical focusing system. The chromium/gold electrodes (10 nm/60 nm) are fabricated by electron-beam lithography (EBL) and electron beam evaporation (EBE), and the channel width between the electrodes is 20 μm. BP flakes selected with appropriate thickness are transferred to the edge of one electrode, followed by the transfer of a MoS_2_ flake of suitable thickness to connect the BP and the other electrode. A larger piece of hBN is finally selected to cover the entire BP-MoS_2_ device. Both BP and MoS_2_ are chosen in thinner flake forms to ensure that the BP-MoS_2_ heterojunction region is completely depleted, thus providing this device with a better response speed. hBN has good light transmittance and is non-conductive, making it an ideal protective layer for this device. To enhance the interfacial properties and the optoelectronic performance of the materials, each type of material undergoes thermal treatment during the transfer process. In this work, characterized by AFM (Supplementary Information Figure S2) the thickness of BP is 24.1 nm, MoS_2_ is 9.1 nm, and hBN is 79.1 nm. This device remains operational and conductive after being placed in the air for three months. Upon our testing, the heterojunction device exhibits good stability, and its dark current, photo response, and temporal response stability are all well-maintained.

To further understand the working mechanism of the van der Waals heterojunction, we drew the band diagram of this device. Figure 1(b) and (c) shows the band diagram of this device, indicating that the device is a Type II heterojunction with good separation efficiency for photogenerated charge carriers [50].

Figure 1(d) shows the IV_d_ curves of the device under 3,390 nm laser. It is worth noting that in the Mid-IR spectrum, the photothermal effect significantly influences the total current [37], [51]. In order to ensure that the photocurrent in this work comes from the photoconductive effect, the IV_d_ curves of in dark and under laser illumination (represented by the black and red lines in Figure 1(d)) are tested. The device was further tested by continuously exposing it to a 3,390 nm laser for 1 min, after which the laser source was immediately turned off and the dark current was measured, as shown by the blue line in Figure 1(d). It can be observed that the device exhibits a certain response to the Mid-IR laser at negative bias, encompassing both photothermal and photoconductive effects, whereas at positive bias, there is almost no response. When the applied bias crosses −0.15 V and +0.1 V, the total current rapidly increases, leaving an approximately flat region near zero bias. In the experiment, to prevent device burnout due to high voltage, we did not test biases voltage with absolute values exceeding 1.5 V. To further verify the impact of the photoconductive effect on the device’s response, we measured time-dependent response curves, which will be elaborated in the following discussion.

To verify the concept of the device, we employed Raman spectroscopy, photocurrent mapping, and AFM for characterization, with the photocurrent mapping results depicted in Figure 1(e)–(g) (the Raman spectroscopy and AFM characterization results will be provided in the Supplementary Information Figure S2). Figure 1(e) is a photograph of the device under an optical microscope, where the orange dashed frame indicates BP, the green dashed frame indicates MoS_2_, the red dashed frame denotes the BP-MoS_2_ heterojunction region, respectively and the white dashed line outlines the electrode edge with a channel width of 20 μm. The annotations in Figure 1(f) and (g) are consistent with those in Figure 1(e). It can be observed that when a negative bias is applied, the heterojunction region exhibits distinct photocurrent behavior as shown in Figure 1(f), and when a positive bias is applied, MoS_2_ displays significant photocurrent behavior as indicated in Figure 1(g), thus demonstrating the device’s responsiveness from negative to positive voltages. The Mid-IR wavelength exceeds the response range of MoS_2_, hence the device shows negligible response under positive bias in the Mid-IR, which is consistent with our subsequent experimental results.

**Figure 2:**
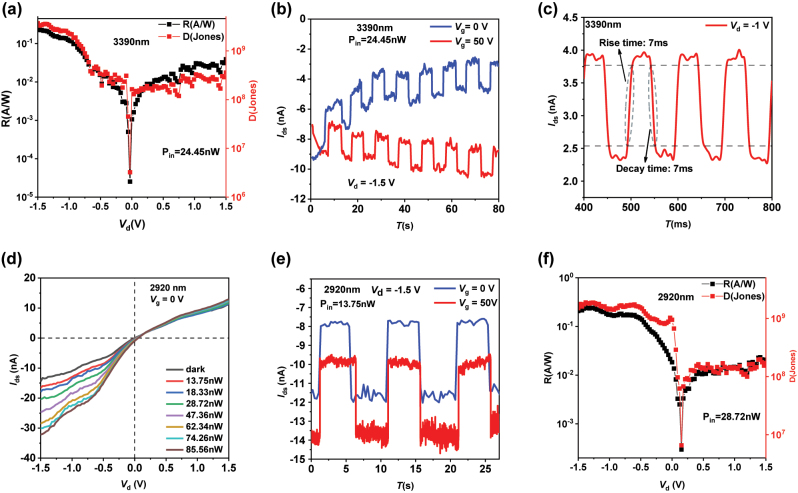
The response characteristics, response speed, and the responsivity (*R*) and specific detectivity (D*) of the device in the Mid-IR band at 3,390 nm. (a) *R* and D* of the device under 3,390 nm laser. (b) The temporal response characteristic curve of the device under 3,390 nm laser. (c) The response speed test results of the device under 3,390 nm laser. (d) The IV_d_ curves of the device under different laser power levels of 2,920 nm laser. (e) The temporal response characteristic curve of the device under 2,920 nm laser. (f) *R* and D* of the device under 2,920 nm laser.

**Figure 3:**
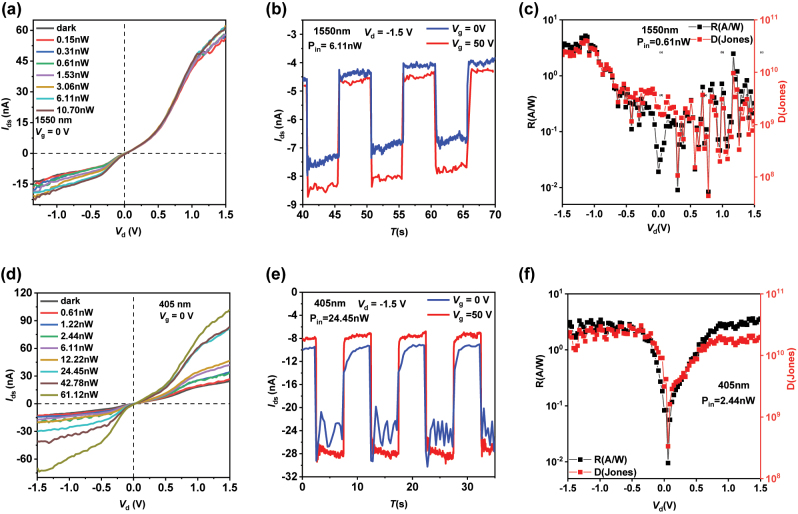
The transmission characteristic curves, temporal response characteristic curves, and the *R* and D* graphs for 1,550 nm and 405 nm laser. (a) The IV_d_ curves of the device under different laser power levels of 1,550 nm laser. (b) The temporal response characteristic curve of the device under 1,550 nm laser. (c) *R* and D* of the device under 1,550 nm laser. (d) The IV_d_ curves of the device under different laser power levels of 405 nm laser. (e) The temporal response characteristic curve of the device under 405 nm laser. (f) *R* and D* of the device under 405 nm laser.

Next, we discuss the IV characteristic curves, temporal response characteristics, and calculate the responsivity (*R*) and specific detectivity (D*) of the van der Waals heterojunction photodetector at different wavelengths. Figures 2 and 3 present the experimental and computational results of the device in the Mid-IR, near IR, and visible light. The laser sources used in the testing conditions are all continuous, and the tested wavelengths are 3,390 nm, 2,920 nm, 1,550 nm, and 405 nm.

For the Mid-IR light at a wavelength of 3,390 nm, due to the limitation that the laser power cannot be adjusted, we only tested the IV curve at a single power level (Figure 1(d)), the temporal response curve (Figure 2(a)), and the calculated results for *R* and D* (Figure 2(b)). To enhance the response of this device at the 3,390 nm wavelength, we focused the spot using a Mid-IR optical microscope system; during the experimental test, the spot diameter was approximately 500 μm. The calculation formulas for *R* and D* are as follows:

$$R=\frac{\left(I_{\text{light}}-I_{\text{dark}}\right)A_{\text{laser}}}{P_{\text{in}}A_{\text{device}}}$$

$$D^{*}=R\sqrt{\frac{A_{\text{device}}}{2eI_{\text{dark}}}}$$

where *I*_light_ is the photo current, *I*_dark_ is the dark current, *A*_laser_ is the spot size of the laser, *P*_in_ is the power of the laser, *A*_device_ is the area of the heterojunction, *e* is the electrical charge.

As shown in Figure 2(a), it can be observed that at an incident light power of 2 mW, the device exhibits its maximum response at −1.5 V, with a responsivity and specific detectivity reaching 0.25 A/W and 3.7 × 10^9^ Jones, respectively. Compared with many previously reported two-dimensional material-based Mid-IR photodetectors, this device demonstrates a distinct performance advantage. Moreover, the device’s responsivity and specific detectivity at negative bias are significantly greater than at positive bias, which further corroborates our previous analysis that the device shows pronounced photocurrent response in the heterojunction region at negative bias, while at positive bias, the response is primarily dominated by MoS_2_.

To elucidate the response mechanism of the device in the Mid-IR spectrum, we measured the temporal response curve of the device under 3,390 nm laser light (Figure 2(b)). As depicted in Figure 2(b), when the Mid-IR light is incident on the device, the material’s self-excitation and lattice vibrations concurrently affect the carrier mobility. Different gate voltages can alter the trend of dark current drift. When a larger gate voltage is applied, the self-excitation effect of the material predominates, increasing the carrier mobility and, consequently, the dark current drift, leading to an upward trend in the total current magnitude. Conversely, when a negative or small gate voltage is applied, the material’s lattice vibrations become dominant, reducing the carrier mobility and causing a downward trend in the total current magnitude. Additionally, within the Mid-IR range, not only the photocurrent generated by the photoconductive effect but also that produced by the photothermal effect is significant, as frequently noted in previous studies. It has been reported in past research that continuous exposure to Mid-IR light can cause a dark current offset due to the photothermal effect, which can account for one-third or even one-half of the total current. In our experiments, as shown in Figure 1(d), under low power and small spot conditions, the dark current drift at 3,390 nm even exceeded half of the total current. Similarly, based on previous studies, pre-irradiation with continuous Mid-IR light for a certain period can stabilize the dark current drift, allowing us to neglect the impact of the photothermal effect in the measurement of the temporal characteristic response curve. Our subsequent test results are all measurements taken after a brief period of pre-irradiation.

Figure 2(c) presents the response speed test curve under 3,390 nm laser, with the testing equipment structure displayed in the Supplementary Information (Figure S3). We utilized a signal generator to convert the continuous 3,390 nm light into frequency-tunable pulsed light and employed a signal amplifier for current amplification. The measurement results indicate rise and decay times of 7 ms, demonstrating a relatively fast response speed compared to some existing two-dimensional material-based Mid-IR photodetectors.

To verify the impact of different laser power levels on the device’s response and detection performance in the Mid-IR spectrum, we selected a 2,920 nm laser as our light source and tested the response curves at seven different power levels, with a spot diameter of 1 cm during the tests. As shown in Figure 2(d), significant current changes were observed in the negative bias voltage region of the device, and almost no significant changes in the positive bias voltage region, which is consistent with the results obtained from our photocurrent mapping and further confirms the conclusions mentioned earlier. Observing the IV_d_ curve in Figure 2(d), the heterojunction exhibits nonlinear behavior; when the bias voltage is less than −0.2 V, a rapid increase in the total current is observed, indicating the generation of photogenerated current. Further observation reveals that when the laser power is high, there is still current at a bias voltage of 0 V, which is due to the current generated by the device’s photoconductive effect and photothermal effect. Figure 2(e) presents the temporal response characteristic curve of the device under an incident light power of 45 nW, showing a pronounced photoconductive effect, with the generated photocurrent being approximately 4 nA. Meanwhile, the gate voltage changes the magnitude of the dark current but hardly affects the value of the photocurrent, which differs from the visible and near-infrared bands. To assess the specific performance metrics of the device, we calculated the voltage-based *R* and D*, with the test results shown in Figure 2(f). To minimize measurement errors, we set the bias voltage to −1.2 V. According to the results in Supplementary Information (Figure S4), *R* and D* increase at lower incident laser powers and exhibit a decreasing trend under higher laser powers. Therefore, we selected an incident laser power of 94 mW, at which the maximum values of *R* and D* in the tested data are 0.223 A/W and 1.74 × 10^9^ Jones, respectively, demonstrating the device’s superior detection performance in the Mid-IR. The *R* and D* calculated based on power are also consistent with the subsequent trends of the visible light (405 nm) and near-infrared (1,550 nm) curves. This may be due to the surface defects of the materials constituting the heterojunction photodetector; when the laser power is low, photons preferentially fill the device’s surface defects, hence *R* and D* both show an upward trend. When the defect filling reaches saturation, the device’s *R* and D* correspond to their maximum values. At the same time, the change in incident light intensity affects the magnitude of the photocurrent [52]. As the light intensity increases, the photocurrent also increases, but the specific detectivity D* typically decreases. This is because, with the increase in light intensity, the effect of dark current relatively diminishes, but the rate of increase in photocurrent is usually slower than the rate of increase in light intensity, leading to a decrease in specific detectivity. To determine the specific saturation point of defect filling in the device, further experimental testing is required.

Following, we discuss the near-infrared laser, for which we selected the 1,550 nm as our experimental test laser source, with the results shown in Figure 3(a)–(c). The laser source used in the experimental test was continuous 1,550 nm light, and the spot diameter during testing was 1 cm. It can be observed that the device also exhibits nonlinear behavior, and its threshold voltage is close to the value obtained in subsequent visible light (405 nm) tests, further confirming the previous test results. Moreover, when a positive bias is applied, the device shows no significant response. This is because the bandgap of typical bulk MoS_2_ is 1.2 eV, corresponding to an absorption edge of 1,033 nm; hence, it has almost no response to 1,550 nm near-infrared laser. This is consistent with our experimental results. After calculation, as shown in Figure 3(c), under a −1.2 V bias voltage, the *R* and D* of the device are 4.8 A/W and 3.68 × 10^10^ Jones, respectively, and the trend is consistent with the test results at 2,920 nm, further proving that material surface defects cause *R* and D* to rise at lower power levels, peak, and then decline. Figure 3(b) is the temporal response characteristic curve of the device under an incident light power of 20 mW. Compared to the Mid-IR band, under 1,550 nm near-infrared laser, the photocurrent will increase to some extent under the influence of the gate voltage, which may be due to MoS_2_ having a certain degree of impact on the device’s response in the visible and near-infrared bands. As depicted in Figure 3(d), the IV characteristic curves of the device under 405 nm continuous laser are presented, with the test variables consisting of 8 different laser power levels, and a spot diameter of 5 mm during the experiment. Under dark conditions, the device exhibits nonlinear transmission characteristic curves and distinct heterojunction behavior is observed. Upon applying 405 nm laser light of varying power, by examining the IV_d_ curves in Figure 3(d), we notice that when the bias voltage crosses −0.2 V and +0.2 V, the absolute value of the total current rapidly increases, leaving an approximately flat region near zero bias. Furthermore, the rate of increase begins to decline after surpassing −0.5 V and +1 V, which may indicate the onset of saturation in the device. Figure 3(e) illustrates the temporal response characteristic curve of the device under 405 nm laser irradiation, showing a significant photo response within the visible light spectrum. Even under a gate voltage of 0 V, the device generates a photocurrent of 15 nA, which further increases to 20 nA under a gate voltage of 50 V. This is approximately 2–3 times the magnitude of the dark current, clearly demonstrating the device’s excellent performance in the visible light region. Subsequently, we calculated the device’s *R* and D* based the experimental data, as shown in Figure 3(f). At an incident light power of 2 mW, the *R* and D* of this device reached 12.4 A/W and 1.02 × 10^11^ Jones, respectively.

**Figure 4:**
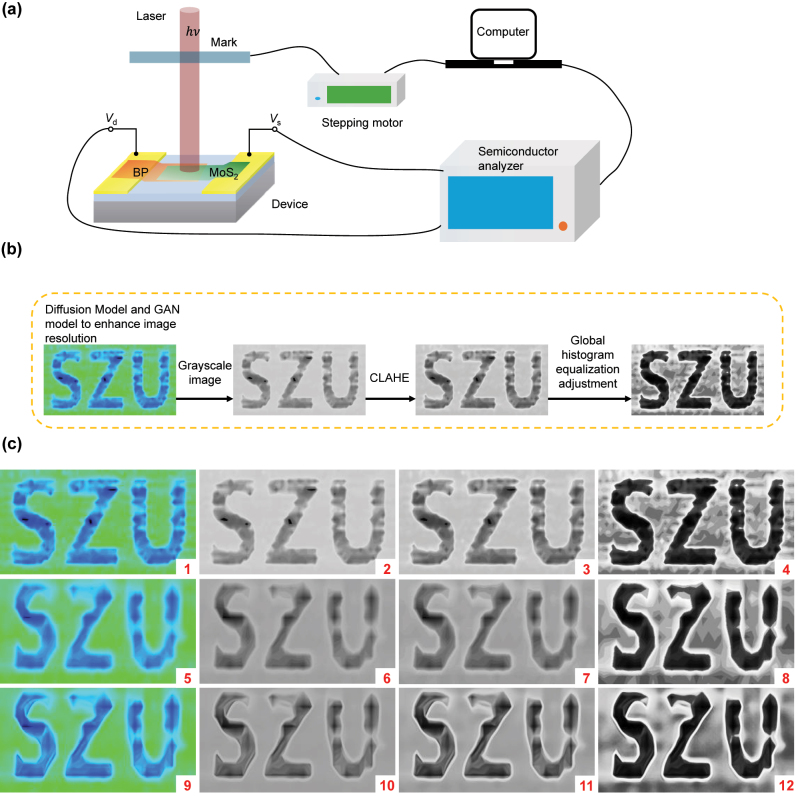
Schematic diagram of the imaging process and imaging results at 3,390 nm. (a) Schematic diagram of the overall structure of the imaging system; (b) flowchart of AI-enhanced imaging quality. (c) Imaging and optimization results, from left to right are the color mapping images, grayscale images, contrast-limited adaptive histogram equalization (CLAHE) images, and global histogram equalization images. Here, pictures No. 1–4 correspond to the original sampling imaging and optimization results, pictures No. 5–8 correspond to the under-sampling imaging and optimization results, and pictures No. 9–12 correspond to the AI-enhanced under-sampling imaging and optimization results.

Finally, we verified the imaging capability of the device using a single-pixel imaging system. The experimental system, as shown in Figure 4(a), consists of a Mid-IR laser source, optical system, Mid-IR photodetector, semiconductor analyzer, mechanical motion system, and control system. Specifically, the Mid-IR laser is focused onto the Mid-IR photodetector via an optical focusing system, and then the photoelectric information of the target object is collected through the semiconductor analyzer. A mechanically controlled movable patterned shield is used to change the object information, and finally, the computer system reconstructs the high-quality object pattern from the collected data using reconstruction algorithms. In this work, the shield pattern is “SZU.” The image quality is mainly influenced by several factors: the power of the laser, the focusing performance of the optical system, the operating speed and accuracy of the mechanical system, the sampling speed and accuracy of the sampling system, and the quality of the pattern reconstruction algorithm. In this work, the dimensions of the shield pattern are 50 mm × 25 mm. During the measurement process, the diameter of the spot is 5 mm. and the distance between the shield pattern and the device is 50 mm, and the laser power is 50 mW.

To facilitate the analysis of image quality, we used the grayscale image of the pattern as the object of analysis, as shown in Figure 4(c) picture No. 1–4. Picture No. 1 is the result obtained by direct color mapping of the collected data, and picture No. 2 is the object grayscale image with a resolution of 730 × 394. The edges of the object are clearly visible, and the “SZU” characters can be clearly recognized. To further improve image quality, we used AI to assist in image optimization and enhancement, as shown in Figure 4(c) picture No. 3. It shows the result of AI-assisted CLAHE image [53]. On this basis, AI is used for global histogram equalization adjustment, as shown in Figure 4(c) picture No. 4. The edges of the pattern are significantly enhanced, and the clarity and recognizability of the “SZU” characters are greatly increased. After calculation, the contrast ratio of the pattern increased from 0.227 (Figure 4(c) picture No. 2) to 0.890 (Figure 4(c) picture No. 4). At the same time, we also analyzed the impact of the size of the histogram equalization region during the optimization process on the final image quality, and this part of the results will be mentioned in the Supplementary Information (Figure S6).

Furthermore, to analyze the optimization results under the condition of under-sampling, we collected data at a sampling rate of 25 % and performed imaging and analysis, as shown in Figure 4(c) picture No. 5–8. It is observable that in under-sampling conditions, even if the image is optimized using the algorithm of CLAHE, the resolution and clarity of the image still have a significant decline, and the edges of the “SZU” pattern are only vaguely visible. To enhance image quality, we used AI to enhance the image resolution. The process is divided into two steps: the first step is to use the Diffusion Model to enhance the resolution of the image [54], [55], to deblur and eliminate noise, and to reduce image defects; the second step is to use the Generative Adversarial Networks (GAN) Model to sharpen the image edges [56], [57], and further denoise and deblur to enhance the overall image quality, as shown in Figure 4(c) picture No. 9. Compared with the original image, the resolution of the enhanced image has reached 2,068 × 1,104, and its clarity has been greatly improved. At the same time, we also used the CLAHE to enhance the image, and the result is shown in picture No. 10–12. In contrast to the enhancement outcomes achieved with under-sampled data, the results after AI enhancement of resolution and image quality are significantly clearer, the overall quality of the pattern is significantly improved, and the shape of the pattern is clearly visible. Through the above imaging experiments and AI optimization results, AI demonstrates significant potential for enhancing image reconstruction within the imaging process. It can stably improve the final image quality under adverse factors such as poor accuracy and slow speed of the sampling system, and lower photodetector performance. This is more economical and effective than traditional single-pixel imaging, and AI can not only be applied to image reconstruction but also to the optimization of the sampling process, to obtain high-quality data more efficiently and conveniently, thereby saving a lot of time and cost.

## 3 Conclusions

In conclusion, we constructed a BP/MoS_2_/h-BN heterojunction Mid-IR photodetector by optimizing the thickness of the heterojunction and the quality of the interfacial region, achieving a wideband response from the visible to the Mid-IR spectrum. At a wavelength of 3,390 nm in the Mid-IR region, the device demonstrated a responsivity of 0.25 A/W and a detectivity of 3.7 × 10^9^ Jones, which is highly competitive among Mid-IR photodetectors. Additionally, the device exhibited a response time of 7 ms in the Mid-IR band. Potential directions for improvement in this work may include enhancing the response speed (by fabricating vertical heterostructures) and increasing the responsivity (such as optical microcavities to enhance the photo response). Building upon these findings, we conducted a Mid-IR single-pixel scanning imaging experiment to validate its potential in imaging applications and further enhanced the imaging results by integrating traditional algorithms with AI. We effectively combined algorithms such as color mapping, CLAHE, Diffusion Model, and GAN Model to achieve high-quality imaging. Under full sampling conditions, the contrast ratio of the imaging results increased from 0.227 to 0.890, and the resolution of the image increased from 730 × 394 to 2,068 × 1,104. Even in under-sampling conditions (at a 25 % sampling rate), high-quality imaging results were still attainable. This underscores the significant potential of AI in the field of Mid-IR imaging, suggesting that it can achieve equal or superior imaging quality while maintaining or reducing sampling time and costs, which in turn could catalyze rapid advancements in the field of Mid-IR imaging.

## 4 Materials and methods

Materials and device fabrication: Cr/Au electrodes were patterned on a 300 nm Si/SiO_2_ substrates by using EBL and deposited using EBE to complete the device structure. Then, BP flakes of appropriate thickness were selected and transferred to the edge of one electrode using a polydimethylsiloxane (PDMS) stamp. Subsequently, MoS2 flakes of suitable thickness were transferred to connect the BP and the other electrode. Finally, a larger piece of hBN was selected to cover the entire BP/MoS2 device. All of these transfer processes has an extra thermal treatment (70 °C) within 1 min.

Characterization: For atomic force microscopy (AFM) measurement, the sample was placed on a platform. Surface morphology data of the sample were then collected, and the 3D surface morphology was reconstructed by a computer. For Raman spectroscopy and photocurrent mapping measurement, the sample was placed on a platform, and corresponding measurements were carried out at room temperature using a ×50 objective. The testing laser had a wavelength of 532 nm with a power of 1 mW. Under ambient conditions, a semiconductor analyzer was used to evaluate the channel current of the device. The device was connected to a preamplifier and a digital oscilloscope to measure its response speed. The spectra of current noise power density for this heterojunction device were measured by a semiconductor analyzer.

## Supplementary Material

Supplementary Material Details
