# Supplementary material for: Artificial intelligence driven Mid-IR photoimaging device based on van der Waals heterojunctions of black phosphorus
Source: Nanophotonics. 2025 Feb 13;14(4):503–13. doi: 10.1515/nanoph-2024-0613 (PMC11834053; doi:10.1515/nanoph-2024-0613)
Supplement: Supplementary file 1 — Supplementary Material Details [file j_nanoph-2024-0613_suppl_001.docx]

**Supplementary Materials**

**Artificial intelligence driven** **Mid-IR photoimaging device based on van der Waals heterojunctions of black phosphorus**

Ziqian Wang, Huide Wang, Chen Wang, Yushuo Bao, Weiying Zheng, Xiaoliang Weng, Yihan Zhu, Yi Liu, Yule Zhang, Xilin Tian, Shuo Sun, Rui Cao, Zhe Shi, Xing Chen, Meng Qiu, Hao Wang, Jun Liu, Shuqing Chen, Yu-Jia Zeng, Wugang Liao, Zhangcheng Huang, Haiou Li, Lingfeng Gao, Jianqing Li^*^, Dianyuan Fan, Han Zhang^*^

**Corresponding authors: Han Zhang,** State Key Laboratory of Radio frequency Heterogeneous integration, International Collaborative Laboratory of 2D Materials for Optoelectronics Science and Technology, Institute for Advanced Study in Nuclear Energy & Safety, Interdisciplinary Center of High Magnetic Field Physics of Shenzhen University, College of Physics and Optoelectronic Engineering, Shenzhen University, Shenzhen 518060, China

Email: [hzhang@szu.edu.cn](mailto:hzhang@szu.edu.cn).

<https://orcid.org/0000-0002-0166-1973>;

**Co-corresponding authors: Jianqing Li,** School of Computer Science and Engineering, Macau University of Science and Technology, Macau,

Email: [jqli@must.edu.mo](mailto:jqli@must.edu.mo).

**Ziqian Wang,** **Rui Cao,** School of Computer Science and Engineering, Macau University of Science and Technology, Macau,

Email: [wangziqian@szu.edu.cn](mailto:wangziqian@szu.edu.cn) (Ziqian Wang), [caorui808@163.com](mailto:caorui808@163.com) (Rui Cao).

**Ziqian Wang, Huide Wang, Yihan Zhu, Yi Liu, Yule Zhang, Xilin Tian, Shuo Sun, Rui Cao,** State Key Laboratory of Radio frequency Heterogeneous integration, International Collaborative Laboratory of 2D Materials for Optoelectronics Science and Technology, Institute for Advanced Study in Nuclear Energy & Safety, Interdisciplinary Center of High Magnetic Field Physics of Shenzhen University, College of Physics and Optoelectronic Engineering, Shenzhen University, Shenzhen 518060, China,

Email: [wangziqian@szu.edu.cn](mailto:wangziqian@szu.edu.cn) (Ziqian Wang), [wanghuide@szu.edu.cn](mailto:wanghuide@szu.edu.cn) (Huide Wang), [zhuyihan0527@foxmail.com](mailto:zhuyihan0527@foxmail.com) (Yihan Zhu), [3034107853@qq.com](mailto:3034107853@qq.com) (Yi Liu), [2060493009@email.szu.edu.cn](mailto:2060493009@email.szu.edu.cn) (Yule Zhang), [txl000522@163.com](mailto:txl000522@163.com) (Xilin Tian), [ssh4543818@163.com](mailto:ssh4543818@163.com) (Shuo Sun), [caorui808@163.com](mailto:caorui808@163.com) (Rui Cao).

**Chen Wang, Meng Qiu,** College of Chemistry and Chemical Engineering, Ocean University of China, Qingdao 266100, China,

Email: [wangchen2518@stu.ouc.edu.cn](mailto:wangchen2518@stu.ouc.edu.cn) (Chen Wang), [mengqiu@ouc.edu.cn](mailto:mengqiu@ouc.edu.cn) (Meng Qiu).

**Yushuo Bao, Jun Liu, Shuqing Chen, Dianyuan Fan,** International Collaborative Laboratory of 2D Materials for Science and Technology of Ministry of Education, Institute of Microscale Optoelectronics, Shenzhen University, Shenzhen 518060, Guangdong, China,

Email: [Baoys1997@163.com](mailto:Baoys1997@163.com) (Yushuo Bao), [liu-jun-1987@live.cn](mailto:liu-jun-1987@live.cn) (Jun Liu), [shuqingchen@szu.edu.cn](mailto:shuqingchen@szu.edu.cn) (Shuqing Chen), [fandy@cae.cn](mailto:fandy@cae.cn) (Dianyuan Fan).

**Weiying Zheng, Wugang Liao,** College of Electronic and Information Engineering, Shenzhen University, Shenzhen 518060, China,

Email: [17725921817@163.com](mailto:17725921817@163.com) (Weiying Zheng), [wgliao@szu.edu.cn](mailto:wgliao@szu.edu.cn) (Wugang Liao).

**Xiaoliang Weng, Yu-Jia Zeng,** Key Laboratory of Optoelectronic Devices and Systems of Ministry of Education and Guangdong Province, College of Physics and Optoelectronic Engineering, Shenzhen University, Shenzhen 518060, China,

Emali: [xiaoliangweng@outlook.com](mailto:xiaoliangweng@outlook.com) (Xiaoliang Weng), [yjzeng@szu.edu.cn](mailto:yjzeng@szu.edu.cn) (Yu-Jia Zeng).

**Zhe Shi,** School of Physics & New Energy, Xuzhou University of Technology, Xuzhou 221018, China,

Email: [laser532@126.com](mailto:laser532@126.com) (Zhe Shi).

**Xing Chen,** School of Electronic Engineering, Chengdu Technological University, Chendu 611730, China,

Email: [chenshin@live.cn](mailto:chenshin@live.cn) (Xing Chen).

**Hao Wang,** State Key Laboratory of Radio Frequency Heterogeneous Integration, College of Mechatronics and Control Engineering, Shenzhen University, Shenzhen, 518060 P. R. China,

Email: [whao@szu.edu.cn](mailto:whao@szu.edu.cn) (Hao Wang).

**Zhangcheng Huang,** State Key Lab of Integrated Chips and Systems, Frontier Institute of Chip and System, Fudan University, Shanghai, 200433, China,

Email: [huangzc@fudan.edu.cn](mailto:huangzc@fudan.edu.cn) (Zhangcheng Huang).

**Haiou Li,** Guangxi Key Laboratory of Precision Navigation Technology and Application, Guilin University of Electronic Technology, Guilin 541004, China,

Email: [lihaiou@guet.edu.cn](mailto:lihaiou@guet.edu.cn) (Haiou Li).

**Lingfeng Gao,** College of Material Chemistry and Chemical Engineering, Key Laboratory of Organosilicon Chemistry and Material Technology, Ministry of Education, Key Laboratory of Organosilicon Material Technology, Zhejiang Province, Hangzhou Normal University, Hangzhou 311121, Zhejiang, PR China,

Email: [gaolingfeng@hznu.edu.cn](mailto:gaolingfeng@hznu.edu.cn) (Lingfeng Gao).

These authors (Ziqian Wang, Huide Wang) contributed equally to this work.

**Figure S1:** BP/MoS_2_ heterojunction transfer process. (a) Gold electrodes and silicon/silicon dioxide substrates. (b) BP transfer process. (c) MoS_2_ transfer process.

**Figure S2:** AFM characterization results of BP/MoS2/hBN. (a) AFM characterization results, where the orange, green, and blue lines represent the sampling ranges of BP, MoS_2_, and hBN, respectively. (b) Thickness diagram of BP. (c) Thickness diagram of MoS_2_. (d) Thickness diagram of hBN. (e) Raman spectroscopy characterization diagram of BP under a 532nm laser. (f) Raman spectroscopy characterization diagram of MoS_2_ under a 532nm laser. (g) Raman spectroscopy characterization diagram of BP/MoS2/hBN heterojunction under a 532nm laser.

**Figure S3:** Schematic diagram of the response speed test experiment, which consists of the following parts: modulated laser, detector, semiconductor analyzer, signal amplifier, and oscilloscope.

**Figure S4:** Graph of responsivity(R) and specific detectivity(D*) based on power. (a) R and D* in 405nm laser. (b) R and D* in 1550nm laser. (c) R and D* in 2920nm laser.

**Figure S5:** (a) The band diagram of the device without laser and gate voltage. (b) The band diagram of the device under mid-infrared illumination and different gate voltage.

**Figure S6:** The impact of the size of the histogram equalization region during the optimization process on the final image quality, within each region, from left to right, they are the grayscale image of the original image, the CLAHE image, and the global histogram equalization image, respectively. (a) The grid size set as 1*1. (b) The grid size set as 10*10. (c) The grid size set as 15*15. (d) The grid size set as 30*30.

**Figure S7:** A schematic diagram of noise testing, the spectra of current noise power density of the device, and R and D* based on noise. (a) Schematic diagram of noise testing, consisted of detector and semiconductor analyzer. (b) Spectra of current noise power density of the device. (c) R and D* in 3390 nm laser. (d) R and D* in 2920 nm laser. (e) R and D* in 1550 nm laser. (f) R and D* in 405 nm laser.
